# Outcomes of Open, Percutaneous, and Closed Treatment of Isolated Mandibular Condylar Fractures

**DOI:** 10.7759/cureus.115947

**Published:** 2026-09-08

**Authors:** Hardeep Tiwana, Harneet Sangha, Dean Kennedy, Abigail Salas, Jeffrey Spiegel

**Affiliations:** 1 College of Medicine, Surgery Department, Washington State University, Spokane, USA; 2 Radiology, Anesthesiology, and Internal Medicine, Elson S. Floyd College of Medicine, Spokane, USA; 3 Otolaryngology, Boston University, Boston, USA; 4 Otolaryngology - Head and Neck Surgery, Boston University, Boston, USA

**Keywords:** closed treatment, facial trauma, hospital readmission, mandibular condylar fracture, open reduction, treatment outcomes

## Abstract

Objectives

Mandibular condylar fractures are common facial fractures, and the optimal balance between open and less invasive treatment remains debated. Readmission provides a reproducible hospital-utilization outcome that can be examined in large administrative datasets. This study aimed to compare 30-day readmission following open, percutaneous, and closed treatment of mandibular condylar fractures (MCF) using national inpatient databases.

Methods

We performed a retrospective database study using the 2016 Kids' Inpatient Database (KID) and the 2019-2020 National Readmissions Database (NRD). KID was used only for supplemental pediatric inpatient characterization and was not used to calculate readmission. The 6,240-case analytic cohort and all 30-day readmission analyses, including the pediatric subgroup analyses, were derived from the 2019-2020 NRD. Isolated MCF were identified with ICD-10-CM (International Classification of Diseases, 10th Revision, Clinical Modification) diagnosis codes, and treatment was classified with ICD-10-PCS (Procedure Coding System) procedure codes as open, percutaneous, or closed. Pearson chi-square tests were used for categorical comparisons, with 95% confidence intervals and Cramer's V reported for effect size. A two-sided p < 0.05 was considered statistically significant.

Results

A total of 6,240 NRD index cases met the study criteria. After correction of denominator and decimal-place transcription errors in the prior version, approach-specific 30-day readmission rates ranged from 4.8% to 8.3% across All Patient Refined Diagnosis-Related Groups (APR-DRG) severity strata. No significant differences were identified among open, percutaneous, and closed approaches within severity strata (all p ≥ 0.711). Age- and fixation-stratified analyses similarly showed no consistent difference between internal and external fixation; pediatric readmission results represent the pediatric subset of the NRD and do not include KID encounters.

Conclusion

No treatment approach demonstrated a consistent 30-day readmission advantage across the examined subgroups. Readmission should be interpreted as a hospital-utilization outcome rather than a direct measure of functional or clinical effectiveness. Treatment selection should remain individualized according to patient and fracture characteristics.

## Introduction

Mandibular condyle fractures (MCF) are among the most common facial fractures, accounting for 18-52% of all mandibular fractures [1-2]. The management of these fractures has been a subject of ongoing debate in the field of oral and maxillofacial surgery. Treatment approaches can be broadly categorized into three groups based on invasiveness and fixation methods: closed reduction, percutaneous fixation, and open reduction with internal fixation (ORIF). Closed reduction, the most conservative approach, involves either non-surgical management or maxillomandibular fixation (MMF) to control mandibular movement [3-4].

Percutaneous fixation is a minimally invasive surgical approach using pins or wires inserted through small skin incisions. ORIF involves direct surgical access to the fracture site, allowing for anatomical reduction and rigid fixation using plates and screws [5-6]. The choice between internal and external fixation further refines the treatment approach. Internal fixation, typically used in ORIF, involves the placement of hardware directly on the bone beneath the soft tissues. External fixation utilizes pins or wires that penetrate the skin and attach to an external frame, although it is less commonly used for condylar fractures. Each approach has its advantages and potential complications. Closed reduction is less invasive but may result in inadequate reduction. Percutaneous and open approaches offer better reduction but carry risks, such as facial nerve injury and scarring [7]. The choice of fixation method impacts stability, infection risk, and the need for hardware removal [8-9].

This study aims to provide an overview of the current treatment modalities for MCFs, highlighting the key differences between closed, percutaneous, and open approaches, as well as internal versus external fixation methods. Understanding these distinctions is crucial for optimizing patient outcomes in the management of these challenging injuries [9-10].

## Materials and methods

Database selection

This retrospective database study used the 2016 Kids' Inpatient Database (KID) and the 2019-2020 National Readmissions Database (NRD) from the Healthcare Cost and Utilization Project (HCUP) [11]. The KID was used only for supplemental pediatric inpatient characterization. It was not pooled with NRD for longitudinal outcomes because the KID does not provide the patient-level linkage required to identify 30-day readmission. The 6,240-case analytic cohort was derived from the NRD 2019-2020, and all readmission analyses, including the pediatric subgroup analysis, used NRD data exclusively. Within the NRD, subsequent hospitalizations were linked with NRD_VisitLink. The databases are de-identified; institutional review board approval and individual informed consent were not required.

Patient selection

Patients were first screened for mandibular fractures using International Classification of Diseases, Tenth Revision, Clinical Modification (ICD-10-CM) diagnosis codes. The NRD analytic cohort was restricted to isolated fractures of the condylar or subcondylar process (S02.610A/B, S02.611A/B, S02.612A/B, S02.620A/B, S02.621A/B, and S02.622A/B). Records containing additional noncondylar mandibular fracture sites, including body, coronoid, ramus, angle, symphysis, alveolar, other specified, or unspecified mandibular fractures, were excluded from the isolated-fracture cohort. Subsequent-encounter, delayed-healing, nonunion, and sequela codes were excluded. Treatment was identified using ICD-10-PCS procedure codes and categorized as open, percutaneous, or closed (Appendix). Records with missing variables required for treatment classification or outcome analysis were excluded. After the application of these criteria and the readmission follow-up eligibility requirement, 6,240 NRD index cases were included in the analytic cohort.

Data analysis

Key study variables included age, fixation type, treatment approach, APR-DRG severity level, and ICD-10-CM/PCS codes. APR-DRG severity was analyzed as level 1 (minor loss of function), level 2 (moderate), level 3 (major), and level 4 (extreme); records without a defined APR-DRG severity category were excluded from severity-stratified analyses. A 30-day readmission was defined as a subsequent inpatient admission associated with the same NRD_VisitLink identifier within 30 days after discharge from the index hospitalization. Because NRD linkage does not extend across calendar years, December discharges were not eligible to serve as index admissions in the 30-day readmission analysis; December encounters could only contribute as readmissions following an eligible earlier index hospitalization. This ensured that each index admission had a complete observable 30-day follow-up window. Readmission was treated as a health-care utilization outcome rather than a direct measure of functional effectiveness. Pearson chi-square tests of independence were used for categorical comparisons. For Table 1, treatment approach was compared within each APR-DRG severity stratum; for Table 2, external and internal fixation were compared within each age and severity stratum. Results are reported with the chi-square statistic, degrees of freedom, two-sided p-value, 95% Wilson confidence interval for the readmission proportion, and Cramer's V as the effect-size measure. Cramer's V ranges from 0 (no association) to 1 (stronger association). A two-sided p < 0.05 was considered statistically significant. Revised table calculations were performed in Python 3.13.5 using SciPy 1.17.0. HCUP survey weights were not applied; results therefore describe the analytic cohort and are not weighted national estimates. The previously reported propensity-score/month analysis was removed because its matching specification could not be reproduced from the retained analytic output; it is no longer referenced in the Abstract, Methods, Results, tables, or Conclusions. Table 3 summarizes baseline characteristics of the NRD analytic cohort.

**Table 1:** 30-day readmission by treatment approach and APR-DRG severity level APR-DRG: All Patient Refined Diagnosis-Related Groups

APR-DRG severity	Approach	Readmissions, n/N	Readmission % (95% CI)	Chi-square (df); p-value	Cramer's V
1	Open	85/1,393	6.1% (5.0-7.5)	0.682 (2); 0.711	0.020
	Percutaneous	11/156	7.1% (4.0-12.2)	—	—
	Closed	7/146	4.8% (2.3-9.6)	—	—
2	Open	112/1,561	7.2% (6.0-8.6)	0.369 (2); 0.831	0.015
	Percutaneous	8/114	7.0% (3.6-13.2)	—	—
	Closed	4/75	5.3% (2.1-12.9)	—	—
3	Open	118/1,469	8.0% (6.7-9.5)	0.013 (1); 0.908	0.003
	Percutaneous	7/91	7.7% (3.8-15.0)	—	—
4	Open	95/1,163	8.2% (6.7-9.9)	0.002 (1); 0.961	0.001
	Percutaneous	6/72	8.3% (3.9-17.0)	—	—

**Table 2:** Readmission rates by approach and APR-DRG severity level APR-DRG: All Patient Refined Diagnosis-Related Groups

Age Group	Severity Level	Fixation Type	Readmissions, n/N	Readmission % (95% CI)	chi-square (df); p value	Cramer's V
Pediatric	1	External	6/98	6.1% (2.8-12.7)	1.56 (1); 0.211	0.073
Pediatric	1	Internal	6/196	3.1% (1.4-6.5)	—	—
Pediatric	2	External	6/163	3.7% (1.7-7.8)	0.14 (1); 0.709	0.022
Pediatric	2	Internal	6/132	4.5% (2.1-9.6)	—	—
Pediatric	3	External	6/82	7.3% (3.4-15.1)	0.02 (1); 0.879	0.010
Pediatric	3	Internal	11/162	6.8% (3.8-11.7)	—	—
Pediatric	4	External	3/68	4.4% (1.5-12.2)	0.11 (1); 0.737	0.025
Pediatric	4	Internal	6/108	5.6% (2.6-11.6)	—	—
Teenage	1	External	6/65	9.2% (4.3-18.7)	0.76 (1); 0.384	0.067
Teenage	1	Internal	6/105	5.7% (2.6-11.9)	—	—
Teenage	2	External	6/79	7.6% (3.5-15.6)	0.52 (1); 0.470	0.051
Teenage	2	Internal	6/118	5.1% (2.4-10.7)	—	—
Teenage	3	External	6/58	10.3% (4.8-20.8)	0.54 (1); 0.461	0.059
Teenage	3	Internal	7/100	7.0% (3.4-13.7)	—	—
Teenage	4	External	3/72	4.2% (1.4-11.5)	0.05 (1); 0.828	0.016
Teenage	4	Internal	4/113	3.5% (1.4-8.7)	—	—
Adult	1	External	6/328	1.8% (0.8-3.9)	0.10 (1); 0.752	0.012
Adult	1	Internal	6/393	1.5% (0.7-3.3)	—	—
Adult	2	External	6/360	1.7% (0.8-3.6)	0.12 (1); 0.727	0.014
Adult	2	Internal	6/295	2.0% (0.9-4.4)	—	—
Adult	3	External	19/308	6.2% (4.0-9.4)	0.20 (1); 0.655	0.017
Adult	3	Internal	26/370	7.0% (4.8-10.1)	—	—
Adult	4	External	37/340	10.9% (8.0-14.6)	0.56 (1); 0.454	0.030
Adult	4	Internal	25/276	9.1% (6.2-13.0)	—	—
Elderly	1	External	6/262	2.3% (1.1-4.9)	0.01 (1); 0.923	0.004
Elderly	1	Internal	6/248	2.4% (1.1-5.2)	—	—
Elderly	2	External	6/275	2.2% (1.0-4.7)	0.10 (1); 0.758	0.013
Elderly	2	Internal	6/328	1.8% (0.8-3.9)	—	—
Elderly	3	External	39/244	16.0% (11.9-21.1)	1.83 (1); 0.176	0.062
Elderly	3	Internal	49/236	20.8% (16.1-26.4)	—	—
Elderly	4	External	67/258	26.0% (21.0-31.6)	—	—

**Table 3:** Readmission rates by age group, severity level, and fixation type APR-DRG: All Patient Refined Diagnosis-Related Groups

Characteristic	Category	Patients (n)	Percent
Age group	Pediatric	1009	16.2%
	Teenage	710	11.4%
	Adult	2670	42.8%
	Elderly	1851	29.7%
APR-DRG severity	1	1695	27.2%
	2	1750	28.0%
	3	1560	25.0%
	4	1235	19.8%
Fixation type	External	3060	49.0%
	Internal	3180	51.0%

## Results

In the treatment-approach analysis (Table 1), the corrected denominators sum to the full NRD analytic cohort of 6,240 cases. At APR-DRG severity level 1, readmission occurred in 85/1,393 open cases (6.1%), 11/156 percutaneous cases (7.1%), and 7/146 closed cases (4.8%) (chi-square(2) = 0.68, p = 0.711). At level 2, rates were 7.2%, 7.0%, and 5.3%, respectively (chi-square(2) = 0.37, p = 0.831). No significant difference was identified at level 3 (8.0% open vs. 7.7% percutaneous; p = 0.908) or level 4 (8.2% vs. 8.3%; p = 0.961). These values correct the denominator and decimal-place transcription errors in the previous version and use the same 30-day readmission definition throughout the manuscript.

Age- and fixation-stratified analyses are shown in Table 2. These analyses also use the NRD 2019-2020 cohort. Pediatric readmission rates therefore represent pediatric patients within NRD and do not include KID encounters. Pediatric readmission rates ranged from 3.1% to 7.3%, teenage rates from 3.5% to 10.3%, adult rates from 1.5% to 10.9%, and elderly rates from 1.8% to 26.0%. The highest observed subgroup rate was 26.0% among elderly patients with severity level 4 treated with external fixation; no internal-fixation comparator was available for that stratum. In all age-severity strata with both fixation groups available, external versus internal fixation was not significantly associated with readmission (all p ≥ 0.176).

**Figure 1:**
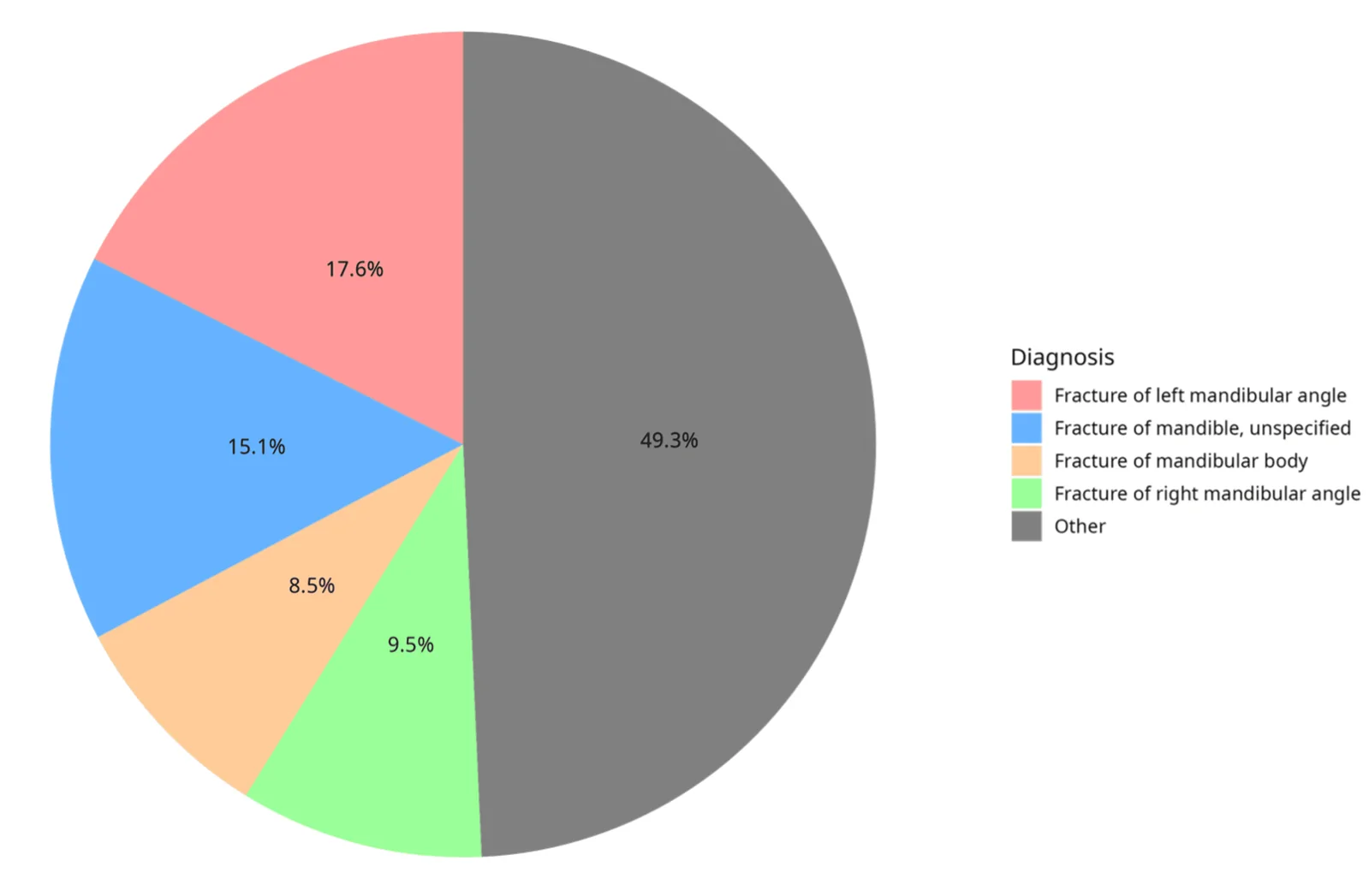
The chart shows the percentages of the top 10 primary diagnoses at readmission following mandibular fracture repair based on ICD-10 codes from the 2020 Nationwide Readmissions Database.

The baseline characteristics of the 6,240-case NRD analytic cohort are summarized in Table 3. Adults comprised 42.8% of the cohort, elderly patients 29.7%, pediatric patients 16.2%, and teenagers 11.4%. Internal fixation accounted for 51.0% of cases and external fixation for 49.0%. APR-DRG severity levels 1 through 4 represented 27.2%, 28.0%, 25.0%, and 19.8% of cases, respectively. The ten most common primary diagnoses recorded at readmission are summarized in Figure 1.

## Discussion

If untreated, MCF can lead to malocclusion, facial asymmetry, impaired mastication, and reduced mandibular range of motion [8]. KID 2016 was used for supplemental pediatric inpatient characterization, whereas all 30-day readmission analyses in this study were performed in NRD 2019-2020. This distinction is particularly important for the pediatric rows in Table 2, which represent pediatric NRD patients rather than KID encounters.

When readmission was compared across treatment approaches within APR-DRG severity strata, no approach demonstrated a statistically significant or consistent readmission advantage. Similarly, external versus internal fixation was not significantly associated with readmission in the age-severity strata for which both fixation groups were available. These findings should be interpreted as comparisons of hospital utilization rather than as proof of equivalent functional outcomes.

Khelemsky and colleagues reviewed the evidence for open versus closed treatment and noted short-term functional advantages of open treatment in selected fractures, while longer-term differences were less consistent [8]. Other studies have also reported early benefits of open treatment for severe fractures. Vesnaver et al. demonstrated improved early clinical and radiographic outcomes using a transparotid open approach, and Weinberg et al. reported favorable early functional measures with open treatment in a controlled clinical study [11-16]. These findings highlight how fracture severity, patient selection, and surgical technique can influence comparisons between treatment strategies.

Conversely, less invasive approaches may reduce risks associated with open exposure, including facial nerve injury and visible scarring [4]. A systematic review by Rai et al. found heterogeneity across studies, with some reporting better functional outcomes after open treatment and others finding no significant differences [12-16]. Variation in sample size, fracture pattern, operative technique, and outcome definition likely contributes to these mixed findings. The present study adds a large administrative-database perspective focused specifically on inpatient readmission rather than detailed functional outcomes.

Age also influences treatment selection. Chrcanovic emphasized the importance of recognizing MCF in children because missed injuries may affect subsequent facial growth [11-13]. Pediatric patients have greater remodeling potential, and open treatment is generally used more selectively in younger patients. Recent systematic reviews and meta-analyses have also demonstrated that ORIF may improve selected functional outcomes, including temporomandibular joint pain, laterotrusion, and maximal mouth opening, while carrying operative risks such as infection [9-10]. Ibrahim et al. reported contrasting findings for some measures of lateral excursion, illustrating that no single outcome uniformly favors one approach [11-14].

In this study, open, percutaneous, and closed treatment did not differ consistently with respect to subsequent inpatient readmission. This narrower finding should not be interpreted as clinical equivalence among techniques. Treatment selection remains dependent on age, overall clinical condition, fracture displacement, dental occlusion, joint function, associated injuries, and surgeon experience [10-14]. Readmission is one component of outcome assessment and should be considered alongside functional, anatomic, and patient-reported outcomes.

This study has several limitations. Its retrospective administrative-database design is subject to coding error, misclassification, missing data, and residual confounding. APR-DRG severity reflects overall illness severity rather than an anatomic classification of condylar displacement. The databases do not provide detailed imaging, occlusion, maximal interincisal opening, mandibular excursion, facial nerve function, scarring, radiographic reduction, or patient-reported quality of life. Accordingly, 30-day readmission is best interpreted as a health-care utilization outcome and not as a proxy for clinical effectiveness. KID and NRD also differ structurally; KID was not used for readmission analysis. In NRD, patient linkage does not extend across calendar years, so December discharges were excluded as index admissions to ensure a complete 30-day observation window. Finally, HCUP survey weights were not applied, so these results describe the analytic cohort rather than weighted national estimates. Future prospective multicenter studies should integrate functional, radiographic, and patient-reported outcomes with healthcare utilization. 

## Conclusions

Our study provides additional national database evidence regarding treatment of isolated MCF. After correction of transcription errors and a consistent definition of 30-day readmission, no open, percutaneous, closed, internal, or external fixation strategy demonstrated a consistent readmission advantage across the examined subgroups. These findings should not be interpreted as evidence that the approaches are functionally equivalent because administrative data cannot capture occlusion, mandibular motion, temporomandibular joint symptoms, facial nerve outcomes, scarring, radiographic reduction, or quality of life. Instead, the results suggest that readmission risk alone should not determine treatment selection. Decisions should remain individualized according to patient age, fracture characteristics, functional impairment, associated injuries, medical complexity, and surgeon judgment. Prospective studies combining clinical and patient-reported outcomes with utilization measures are needed to better identify which patients benefit most from each treatment strategy.

## Appendices

**Table 4:** ICD-10 (International Classification of Diseases, 10th Revision) conditions and procedural codes.

Category	ICD-10-CM / ICD-10-PCS Codes
Condylar process, unspecified side (include)	S02.610A, S02.610B
Condylar process, right (include)	S02.611A, S02.611B
Condylar process, left (include)	S02.612A, S02.612B
Subcondylar process (include)	S02.620A/B, S02.621A/B, S02.622A/B
Noncondylar mandibular fracture sites (exclude)	S02.60-, S02.63-, S02.64-, S02.65-, S02.66-, S02.67-, S02.69-
Open	0NST0, 0NST04, 0NST04Z, 0NST05, 0NST05Z, 0NST0Z, 0NST0ZZ
Percutaneous	0NST3, 0NST34, 0NST34Z, 0NST35, 0NST35Z, 0NST3Z, 0NST3ZZ, 0NST4, 0NST44, 0NST44Z, 0NST45, 0NST45Z, 0NST4Z, 0NST4ZZ
Closed	0NSTX, 0NSTXZ, 0NSTXZZ
